# Supplementary material for: Mass Spectrometry Imaging of Flavonols and Ellagic Acid Glycosides in Ripe Strawberry Fruit
Source: Molecules. 2020 Oct 9;25(20):4600. doi: 10.3390/molecules25204600 (PMC7587173; doi:10.3390/molecules25204600)
Supplement: Supplementary file 1 [file molecules-25-04600-s001.pdf]

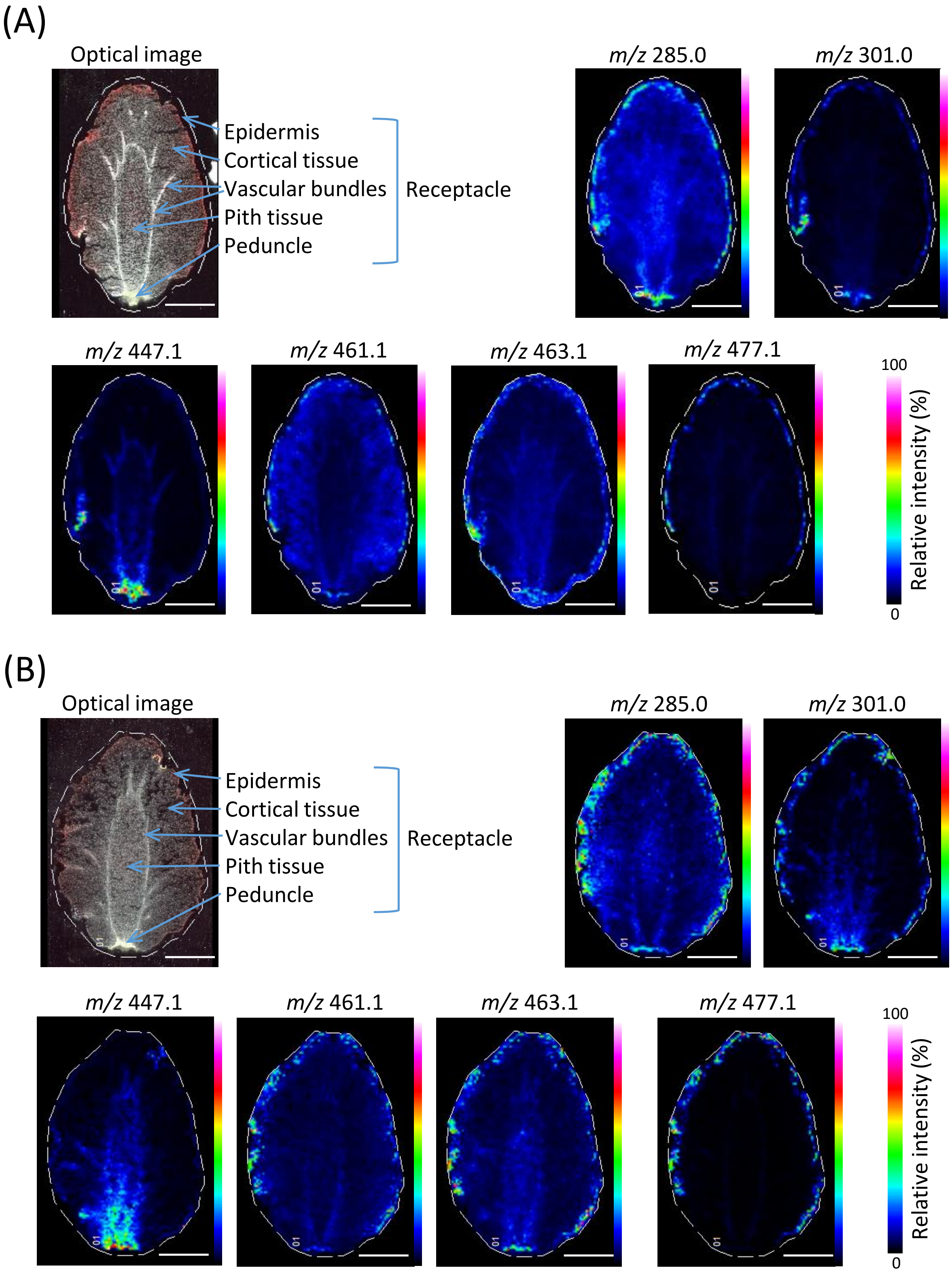

Supplementary Figure S1. Representative ion images of the tentatively identified flavonols and ellagic acid glycosides in strawberry fruit sections by matrix-assisted laser desorption/ionization-mass spectrometry imaging. (A), and (B) These ion images were obtained from two strawberry fruit samples different from that shown in Figure 2. The dotted white line shows the analyzed region. Scale bar = 5 mm.
